# Report of Diseases from September 24th to October 24th

**Published:** 1814-11

**Authors:** John Want

**Affiliations:** North Crescent


### Medical and Physical Journal.

5 OF VOL. XXXII.] NOVEMBER, 1814. [NO. 189.

"For many fortunate discoveries in medicine, and for the detection of numerous errors, the world is indebted to the rapid circulation of Monthly Journals; and there never existed any work to which the faculty in EUROPE and AMERICA were under deeper obligations than to the Medical and Physical Journal of London, now forming a long, but an invaluable series."—RUSH.

### *For the Medical and Physical Journal.*

### REPORT *of* DISEASES *from* SEPTEMBER 24*th to* OCTOBER 24*th*; BY MR. WANT.*

TOWARDS the latter end of the last month, SCARLATINA made its appearance, with symptoms in many cases of a threatening nature. In some it was accompanied by typhus fever; but one instance only of death has occurred within my observation.

MEASLES are also very prevalent. I am informed that several children have died, but as it has been among the poor, in situations unfavourable to recovery, under circumstances otherwise propitious, the fact does not lead to the inference of malignity in the disease.

Strong prejudices still exist, even in well-informed circles, in favour of keeping these patients in a warm temperature. But the effects of a contrary treatment have been again witnessed in several instances; cold ablution of the skin having produced a rapid diminution of the fever, and all the symptoms.

In my own family, where accident furnished me with the means of judging by contrast, the most prejudiced mind must have been satisfied of the superiority of the refrigerant treatment to that generally employed. I was led to adopt this practice three years since, in consequence of observing in my own person the beneficial effects of cold air and large draughts of iced water, in a catarrh of such severity as to confine me to the room, until a professional engagement compelled me to go abroad. The weather was intensely cold, and the relief I experienced in the open air was so remarkable, that upon that occasion I made several experiments on myself, to determine the agency of cold and heat in this *inflammatory* disease.

* For the Meteorological Report, and Remarks on the Weather, see the last pages.

A great weariness, with aching of the limbs, very common in catarrh, induced me to preserve a recumbent position: for the sake of trial, therefore, I went to bed, wrapped my head in flannel, and took warm drink. The temporal arteries immediately began to pulsate, accompanied with other marks of high arterial action about the head and eyes, and a no less prominent effect was an increase of all the catarrhal symptoms. On again quitting the room for the open air, the catarrh abated, but was uniformly increased on my sitting near the fire, or on my return to bed, to which I felt myself inclined from the weariness of my limbs.

The establishment of this fact is important, as it leads to a valuable improvement in the treatment of inflammatory complaints in general. It is admitted, that any thing which accelerates the circulation is hurtful in these affections, yet warmth and warm drinks are considered indispensible to success, and the Systematic instructs us to avoid the use of cold water, as a destructive poison. This prohibition will be found on examination to be not only erroneous, but contrary to the authority of names high in the estimation of the profession. Can the practical precept of Sydenham have escaped observation? With him the most approved modes of treatment, the most copious abstraction of blood, often failed, while the patient was kept to his bed.—Let him speak for himself: "Facio ut lecto quotidie eximatur, idque ad horas aliquot, prout vires suaserint; quod quidem tanti est in hoc morbi genere, ut si lecto æger jugiter affigatur, neque hæc tam larga sanguinis evacuatio, neque remedia alia utcunque refrigerantia, at dicta symptomata perdomanda vel minimum aliquando proficiant."*

Catarrhal and Pneumonic Complaints made their appearance in a few instances about the 5th of October. One case of inflammation of the lungs was fatal, and ran its course with a rapidity truly awful. The subject of it, Mary Keele, 9, Colonnade, Guildford-street, seemingly about ten years of age, was not thought sufficiently well on Sunday to attend a religious school to which she belonged, but was not brought to me until the morning of Tuesday. She was able to walk to and from my house in North Crescent, though she felt great weariness. Her respiration was hurried, and she was troubled with a distressing cough. The sensations about the chest were described as a feeling of constriction rather than actual pain. She was unable to take a full inspiration without an increase of the symptoms. The pulse was quick and feeble; the tongue furred, but no febrile heat on the skin was present.

* Sydenham, op. Pleuritidis curatio.

On

On account of the youth of the girl, and the weakness of pulse, I ordered blood to be taken by cupping from the chest, but for trifling reasons, uninteresting to the public, it was not done, the mother promising most faithfully to let me hear from her in the evening, lest, as I expressed a fear, the symptoms should increase. I saw nothing of her until Thursday, when I was requested to visit her at home. The days lost were fatal to our hopes. I found the respiration had become considerably more laborious, with great anxiety of the countenance. The lips were livid, and she might literally be said to be struggling for breath. The cupping was immediately performed, and a blister applied, but it was too late: in the evening she died.

A delicate female, twenty-two years of age, after having had one child, was afflicted for several months with a discharge of bloody fluid from the uterine organs, accompanied with a train of dyspeptic and hysterical symptoms. The frequent dependence of some species of this complaint on the disordered condition of the stomach, is not sufficiently attended to in practice, which is improperly considered to be the effect instead of the cause. Hence it is usual to order the patient to be kept cool, to live abstemiously; and sometimes bleeding has been most unaccountably recommended even in this species. A variety of treatment had been adopted here without benefit: *bleeding* and cold injections to the vagina had not been forgotten. I prescribed 15 drops of the liquor potassæ subcarbonatis three times a-day, with no other remedy but the occasional use of good wine. This had, as usual, the effect of diminishing the dyspeptic feelings, and the uterine complaint gradually ceased. Reasoning upon some of the effects of this medicine, it would not be easy to account for the benefit arising from it in these cases. Alkalies are known to have the property of attenuating the blood, and the continued use of them, within my own experience, has superinduced scurvy. I should here observe, that its use in menorrhagia has been confined to that form of it which was observed in the patient above-mentioned; and that perhaps in strict propriety the correctness of the term is questionable. Change the colour of the discharge, and the disease would be leucorrhœa.*

Leprosy occurred in a boy eleven years of age. He was literally covered from head to foot, leaving scarcely space sufficient to preserve the characteristic rotundity of

* Cullen calls leucorrhœa menorrhagia alba, for, says he, leucorrhœa almost always either follows or accompanies menorrhagia, and often arises from the same causes as the menorrhagia rubra.

 the the crusts. The appearance of the spots on the face was somewhat different from those in the limbs, being much smaller, and not raised from the level of the surrounding skin, while in the thigh, arms, and particularly in the hairy scalp, a thick incrustation had formed. The Plummer's pill, with an electuary of crude antimony, black sulphuret of quicksilver, and nitre, was given, with evident amendment of the general health; but as it was thought a good opportunity to make trial of different external remedies, to discover their comparative efficacy, it is not easy to say whether it would alone have been sufficient for the cure.

The ointment of nitrated quicksilver, and a saturated liniment of borax and honey, were each applied to different limbs. The face was smeared with a mixture of equal parts of sulphuric acid and spirit of wine, and the right arm was immersed in water as hot as could be borne several times in the day. The sulphuric acid was entirely useless, on the contrary the borax liniment seemed the most eminently beneficial, the crusts being not only more readily loosened by it, but the diseased surface beneath sooner recovered a healthy condition. It was, however, less serviceable when applied to the head, where the crusts were remarkably thick; but an ointment made of slaked lime and lard, gradually removed the disease in this part. The ointment of nitrated quicksilver, and the fomentations of hot water, were also useful, but not in an equal degree. The result of these trials is decidedly in favour of the borax liniment.

Whilst prosecuting the inquiry, I found that an ointment of salt prunella was strongly recommended by the Hon. Mr. Boyle as a specific for leprosy, which, from its resemblance to borax, I am induced to think well of. It should be observed, that in former cases the internal use of the solution of arsenic has generally been followed by complete success, and was laid aside on the present occasion merely for the purpose of making trial of other remedies.

An attentive examination of this case, has strengthened an opinion which I have entertained, that Lepra and Psoriasis (taken in its modern acceptation) are diseases not essentially different. The spots on the face of this subject, had they been found unconnected with the disease, as it shewed itself on the surface of the body, would, according to Willan, have been denominated Psoriasis Guttata; distinguishable only from Lepra (which is said by this author never to appear in the face) by its scale or scales being thinner, and more easily separable than in the latter. In this same case, though in general the leprous crusts were distinct, yet in some parts at the elbows they ran into one another, putting on the character of the Psoriasis Diffusa; and, had the disease been confined to this part, it must have obtained that appellation. Again, at one period, when it was nearly subdued, the spots, originally as large as a sixpence, had progressively lessened, still preserving a circular form, but most of them had lost the thickened crust. The disease in this stage, without consideration of the previous circumstances, would have been called Psoriasis Guttata.

Psoriasis Diffusa, supposed by some to be peculiar to washerwomen, was observed in a very respectable lady in advanced years. A solution of arsenic twice a-day, with Plummer's pill at night, and an ointment, consisting of two drams of sulphuret of potash, and an ounce of lard, are being used with success, as the disease is evidently on the decline.

A melancholy case has been communicated of death occurring soon after parturition, without any symptoms having existed during life sufficient to lead to an apprehension of danger. The labour, of six hours duration, proceeded naturally, if we except a deficiency of effective bearing-down pains, which made it necessary, in the opinion of the attending practitioner, to have recourse to the blunt hook for the extraction of the fœtus. The breech presented, and the delivery was easily effected, but the powers of life gradually failed, and the patient died apparently from exhaustion. On dissection, it was found the cervix uteri and one of the fallopian tubes were in a state of sphacelus; the latter of these was considerably enlarged and tumid. It seems obvious that we must look beyond the process of parturition for the mysterious event of this case. It was recollected that, for about three weeks before delivery, sensations were complained of in the uterine region, which were described as being uneasiness rather than acute pain, and are so commonly felt in similar circumstances that no suspicion was excited of the mischief that was going on; nor were they of sufficient magnitude to prevent the patient's taking her usual walks until the day of her death.

We regret that we have no authority to publish the name of the very respectable accoucheur who attended, the account having been given us without any view to publication.

A wretched receptacle of poverty and filth, in Eagle-street, furnished two cases of Typhus, but they yielded readily to cold ablution. It is hardly necessary to remark, that, independent of the advantage of cleanliness among the poor, washing of the body is extremely useful in all cases of fever, where the heat of the body is above the natural standard. The head-ach, in one of the cases, was intense, and was much relieved by the application of wet cloths to the forehead.

11, *North Crescent.* JOHN WANT.

